# Observability of gustatory information in scalp EEG

**DOI:** 10.1038/s41538-026-00957-7

**Published:** 2026-07-07

**Authors:** Yifei Zhang, Tianyu Dong, Yunhao Gong, Xinyu Wang, Zhongren Wei, Xianghao Wu, Weijia Li, Hongwei Yu, Jingjing Liu

**Affiliations:** School of Automation Engineering, Northeast Electric Power University, Jilin City, China

**Keywords:** Biological techniques, Computational biology and bioinformatics, Neuroscience

## Abstract

Compared with vision and audition, gustation yields weaker and more confound-sensitive scalp (EEG) responses. Here we review gustatory EEG through an observability framework comprising detectability, reproducibility and interpretability. Low-frequency, especially delta-band, activity currently provides the strongest evidence for taste-identity detectability, whereas event-related potentials (ERPs) evidence is stronger for intensity. Spatial and network features remain promising but methodologically constrained. We outline best-practice workflows for cumulative, interpretable gustatory EEG evidence.

## Introduction

Gustation plays a central role in food choice, nutrient intake and risk avoidance^1^. Yet, from the perspective of non-invasive neuroimaging, evidence for the temporal dynamics and representational structure of cortical gustatory processing in scalp EEG has long remained weaker than that for vision and audition. Early studies reported gustatory event-related potentials (gERPs), but substantial differences in stimulus delivery, temporal alignment, participant-state control and analysis pipelines have produced a characteristic situation in which signals can be observed but remain unstable, conditions can be separated but remain difficult to interpret, and findings remain hard to align and compare across studies.

Classical taste research has focused mainly on receptors, ascending pathways and psychological dimensions^2–8^, explaining how chemical stimulation is transduced into neural signals and how basic taste qualities such as sweet, sour, bitter, salty and umami are mapped onto subjective experience^9,10^. These studies provide an essential foundation for understanding the gustatory system, but they do not directly answer a methodological question that is central to scalp EEG: which components of gustatory information can be stably observed and reliably separated? Even when differences are observed, it is often difficult to determine whether they reflect gustatory representations themselves or instead arise from alignment error, movement-related somatosensory inputs, or analysis strategy.

To move beyond the question of whether an effect exists and towards the question of whether that effect is credible, we propose an observability framework that decomposes the evidential strength of gustatory information in EEG into three linked requirements. Detectability concerns whether gustatory stimulation can generate stable and measurable electrophysiological markers under reasonable recording quality and temporal alignment. Reproducibility concerns whether these markers retain consistent direction and magnitude across participants, laboratories, stimulus sets and processing pipelines. Interpretability concerns whether these markers remain present after controlling for confounders and whether they are consistent with gustatory pathways and plausible cortical processing mechanisms.

Within this framework, the present article integrates evidence in three ways. First, by combining gustatory pathways with principles of EEG signal generation, we define physiological observability boundaries and clarify which nodes are more likely to project to the scalp, and which are more likely to appear only indirectly through cortical projections or to be obscured in scalp EEG. Second, we integrate evidence across feature domains by comparing the support provided by time-domain, frequency-domain, spatial and network features for taste identity, intensity and pleasantness, and by summarising their evidence levels. Third, we treat decoding models as information probes, linking model-structure assumptions to interpretable validation so as to construct an inference chain from separability to credible representations.

This article is a narrative review and perspective informed by a structured literature search, rather than a formal systematic review or meta-analysis. Representative studies were identified primarily through Web of Science, PubMed and Google Scholar using combinations of keywords including gustation, taste, EEG, ERP, time-frequency, delta, source localisation, connectivity and decoding. We prioritised human EEG studies that were directly relevant to detectability, reproducibility and interpretability, while selected fMRI and animal studies were included when additional support was needed for source-level or network-level interpretation. This strategy was intended to enhance the transparency of evidence synthesis while preserving the conceptual focus of the observability framework.

On the basis of this synthesis, we further propose an actionable best-practice checklist and briefly discuss possible future applications of gustatory EEG.

Our aim is not to exhaustively catalogue all available literature, but to provide a reusable methodological approach for non-invasive neuroimaging research. Under conditions of low signal-to-noise, deep and folded cortical generators, strong confounds and substantial inter-individual variability, we ask how gustatory EEG can be advanced from scattered observations into a comparable and cumulative evidence base.

## Gustatory pathways and observability boundaries from an EEG perspective

The anatomical route from peripheral taste receptors to the cortex is relatively well defined, but activity along this pathway is not projected to the scalp in proportion to its biological importance. For EEG, whether a node is observable depends not simply on whether it is active, but on whether it can form a macroscopic dipole that is sufficiently large in spatial extent, sufficiently synchronous across trials and sufficiently consistent in orientation, while still preserving a measurable net potential after volume conduction and skull filtering. Distinguishing this point is essential for avoiding two common misinterpretations: treating invisibility as the absence of activity, and treating visibility as direct evidence for a local source.

**Fig. 1:**
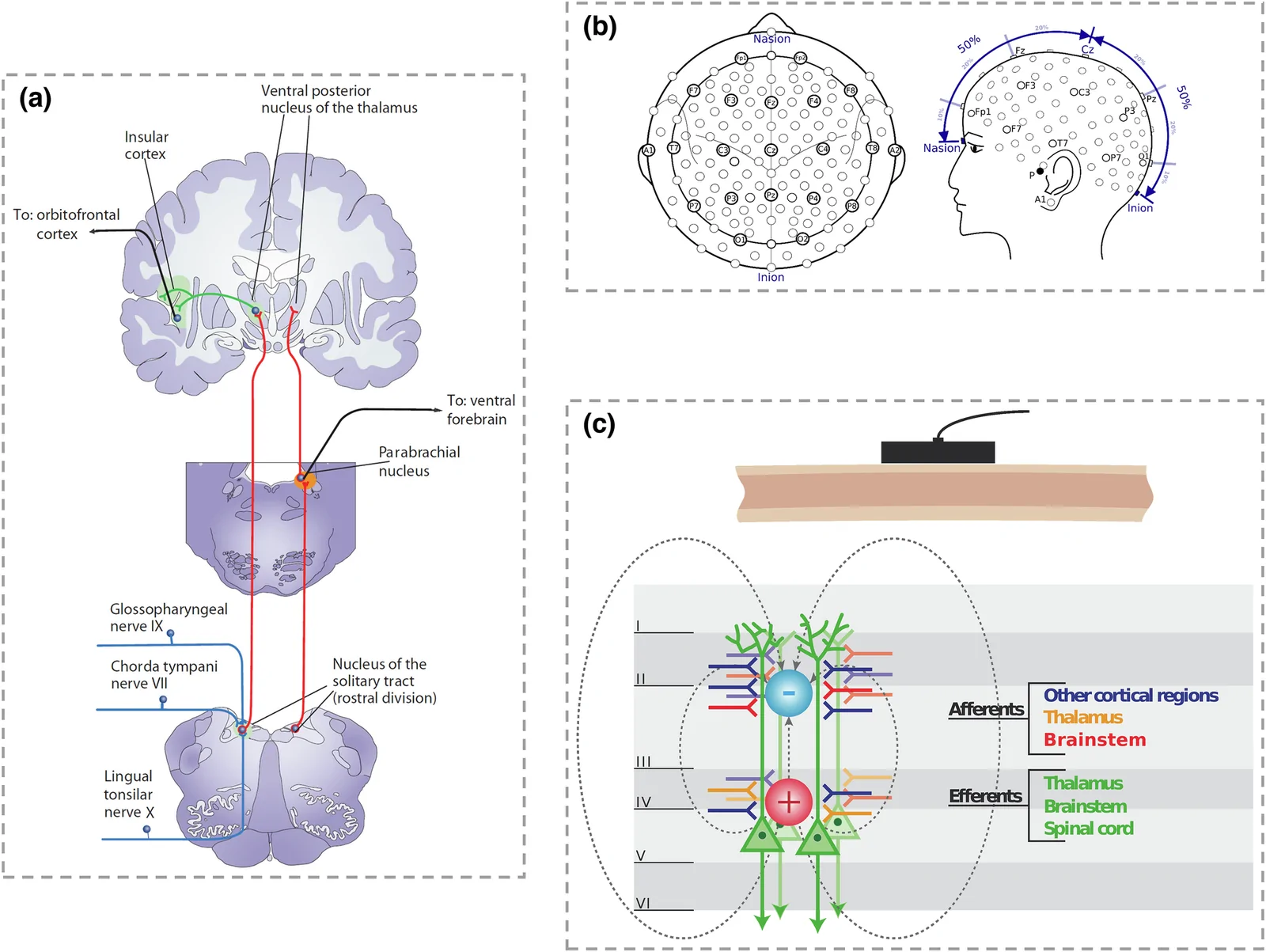
Transmission pathways and measurement of EEG signals. **a** Transmission pathway of gustatory information^23^. **b** Electrode distribution for EEG acquisition^24^. **c** Principles of EEG acquisition^24^.

Gustatory signals travel via the facial nerve (VII), glossopharyngeal nerve (IX) and vagus nerve (X) into the nucleus of the solitary tract (NST) in the medulla^11–13^, then ascend to the ventroposteromedial parvicellular nucleus of the thalamus (VPMpc), and finally project to primary gustatory cortex centred on the insular cortex and frontal operculum^14,15^ (Fig. 1a). From the perspective of observability, the insula–operculum complex is a plausible cortical contributor because it contains large populations of relatively aligned pyramidal neurons. By contrast, NST and VPMpc are small, deep and geometrically complex, and their direct contribution to scalp EEG is usually weak; they are more likely to exert indirect effects through modulation of cortical input. Neuroimaging studies likewise show stable activation at the insula–operculum junction during gustatory stimulation^16^, supporting a cortex-first strategy when searching for robust gustatory EEG markers.

EEG mainly reflects field potentials generated by synchronous postsynaptic activity in large neuronal populations^17,18^, with dendritic currents in cortical pyramidal neurons making a particularly important contribution^19^. Only when population activity forms an open field and yields a coherent dipole are measurable net potentials likely to appear on the scalp^20^. If current orientations are dispersed or locally closed, macroscopic signals tend to cancel and become difficult to observe^21,22^. In addition, dipole-field strength decays rapidly with distance, while the volume conduction properties of the skull and scalp further disperse and attenuate deep sources. Thus, when considered together with electrode geometry and recording principles (Fig. 1b, c), even highly active deep structures often fail to produce stable and localisable waveform components in conventional scalp EEG^23,24^.

Source orientation also shapes observability. EEG is sensitive to both radial and tangential sources^25^. Thus, even though the insula and operculum lie deep within the Sylvian fissure and are often dominated by tangential components, they can, in principle, still contribute to scalp EEG. However, the complex folding of the insular cortex makes partial cancellation between neighbouring gyri and sulci likely, thereby reducing net signal strength and amplifying inter-individual variability^26,27^. More importantly, sensor-level observations should not be equated directly with source-level explanations. Fronto-central scalp projections may be consistent with insula–operculum contributions, but under conventional-density scalp EEG, they do not uniquely establish insula-related activity, especially when somatosensory, motor and swallowing-related signals overlap. Accordingly, source-level interpretations should be considered most credible only when they are supported jointly by high-density EEG or MEG, individualised head models, anatomically constrained inverse solutions, and sensitivity analyses across alternative source priors.

Available empirical results as a whole support a predominantly cortical origin of gustatory EEG. Source-localisation and cross-modal comparison studies report activity peaks in bilateral insula and operculum at approximately 100–160 ms after gustatory stimulation, corresponding to early EEG components^28^. Other electrophysiological imaging work similarly supports early cortical responses in the range of approximately 70–170 ms^29^. At the sensor level, gustatory evoked potentials are typically more pronounced over fronto-central electrodes and often show a midline projection pattern^30^, consistent with the volume-conducted projection of insula–operculum sources.

At the same time, it is important to clarify why insular and opercular regions appear more difficult to observe stably in gustation than in some other sensory modalities. Existing MEG work shows that, under appropriate conditions, the insula can serve as a detectable generator of evoked responses in somatosensory paradigms^31^. Thus, the issue is not that the insula is intrinsically invisible, but that gustatory-specific responses are embedded in an experimental context that is especially vulnerable to confounds. Liquid delivery introduces uncertainty between trigger time and true effective onset. Dissolution and diffusion, saliva mixing, and tongue-surface distribution increase temporal jitter. Gustatory stimulation is often accompanied by tactile, thermal, trigeminal and oral-motor inputs. In addition, swallowing, tongue movements and perioral muscle activity contaminate the same time window in which gustatory responses are expected, thereby reducing the amount of clean data available for averaging and modelling. Together, these factors reduce effective synchrony and effective signal-to-noise ratio, making activity from the gustatory cortex harder to isolate reliably at the scalp, even if the insula–operculum complex does indeed participate in taste processing.

In summary, only neural activity that is sufficiently large in extent, sufficiently synchronous and sufficiently consistent in orientation can overcome the scalp barrier and become visible in EEG. Brainstem and thalamic nuclei within the gustatory pathway are usually too small and insufficiently synchronised to meet this condition. By contrast, the primary gustatory cortex contains large populations of pyramidal neurons that can, when coherently engaged, generate measurable scalp potentials. This helps explain why scalp EEG mainly reflects cortical activity and is often insensitive to deep structures. Although recent studies have attempted to explore subcortical signals such as thalamic or hippocampal activity using high-density EEG and advanced algorithms, the contribution of deep gustatory nodes to scalp EEG is still likely to be negligible under conventional experimental conditions. Thus, the structures that EEG is best suited to observe remain those large-scale synchronously activated cortical nodes along the gustatory pathway. At the same time, making gustatory cortical activity genuinely observable will require not only denser recordings and more refined source modelling, but also improvements in synchronisation, trial-number design and confound control. This provides a firmer basis for interpreting gustatory EEG components and defining practical observability boundaries.

## Evidence across feature domains and evidence grading

The observability of gustatory information is not a single metric. The same stimulus may appear weak in the time domain but stronger in the frequency domain; spatial features may suggest a common origin but differ across participants; and network synchrony may be theoretically relevant, while direct evidence remains limited. To avoid drawing global conclusions from any one class of features alone, we organise the available studies into a variable-by-feature evidence matrix (Table 1) and use four levels—strong, moderate, weak and insufficient evidence—to summarise observability. Here, strong evidence refers to results that are directionally consistent across studies and paradigms and are supported by at least some degree of interpretability; moderate evidence refers to convergent findings that remain sensitive to paradigm details or inter-individual variability; weak evidence refers to scattered findings that depend on specific pipelines or lack stringent validation; and insufficient evidence indicates that relevant signals may exist but current evidence is inadequate to support a reliable conclusion. Importantly, in some cases, evidence levels should be stated separately for detectability and interpretability, because a feature may be relatively stable in separability while still remaining sensitive to confounds.

**Table 1:** Overview of evidence strength for gustatory-variable encoding across EEG feature domains

Domain	Taste identity	Intensity	Pleasantness
Time domain	Weak evidence: early morphologies are similar across tastes, with only occasional differences in later components.	Moderate evidence: ERP amplitude and latency vary with concentration; higher concentrations often increase amplitude and shorten latency, although findings are not fully consistent across studies.	Weak evidence: early components show little sensitivity to subjective liking; later cognitive components, such as P3, may reflect pleasantness differences, but not robustly.
Frequency domain	Strong for detectability; moderate for interpretability: delta-band activity carries highly discriminable identity-related information, but interpretation remains sensitive to slow drifts and movement-related confounds unless swallowing control, EMG/EOG mitigation, matched control conditions and validation strategy are explicitly reported.	Moderate evidence: low-frequency power increases with stimulus intensity and higher concentrations often induce stronger alpha-band suppression.	Weak evidence: delta activity appears to reflect physical taste identity more strongly than pleasantness; alpha- and beta-band effects may contribute, but direct and reproducible evidence remains limited.
Spatial	Weak evidence: taste-related differences in scalp topography are subtle and often require statistical or source-level analysis; sensor-level patterns do not uniquely establish insula-related generators.	Weak evidence: intensity mainly affects amplitude within activated regions rather than consistently changing the set of regions recruited.	Insufficient evidence: no consistent results show reproducible spatial-pattern differences across pleasantness levels.
Network and connectivity	Weak evidence: animal and imaging work suggests network-level structure for identity, but direct human EEG evidence remains limited; reproducibility may improve with source-space, leakage-aware metrics and higher-resolution analyses.	Insufficient evidence: broader synchrony with higher intensity is plausible, but targeted EEG evidence remains sparse.	Insufficient evidence: relevant network effects may exist, but current EEG evidence is limited and not yet reproducible.

At the variable level, taste identity, intensity and pleasantness do not occupy the same evidential level. Taste identity depends more on rapid perceptual categorisation, intensity is closer to continuous magnitude scaling, and pleasantness is more strongly modulated by context, expectation and learning. Because alignment jitter, confounds and observability boundaries compress these variables to different degrees, these differences in hierarchy are further amplified in scalp EEG.

**Fig. 2:**
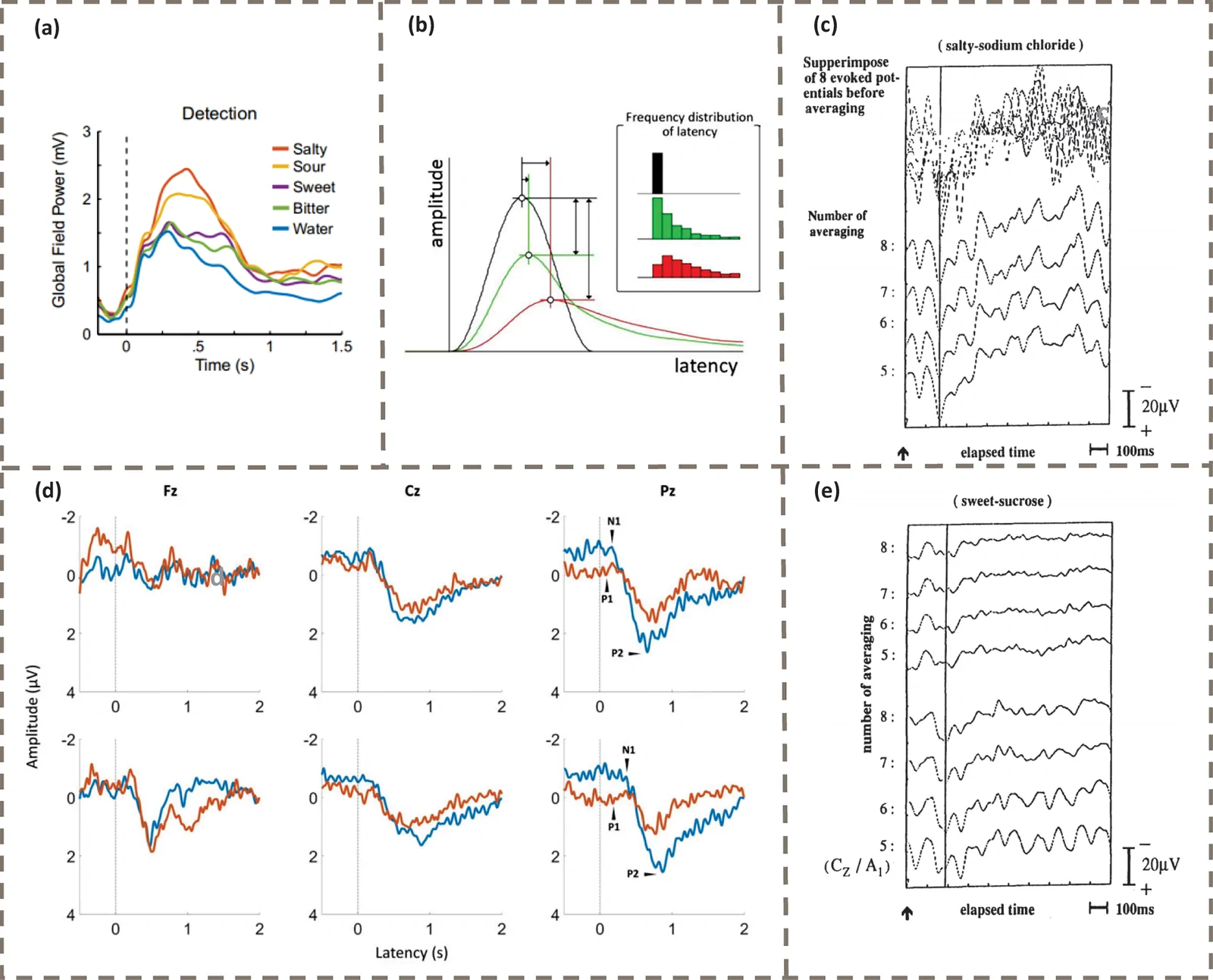
Evidence for time-domain feature-based discrimination. **a** Grand-average GFP time series shows that all basic tastes elicit stable gustatory responses^48^; **b** even when single-trial amplitudes remain unchanged, trial-to-trial latency jitter can distort the averaged ERP by reducing peak amplitude and shifting peak latency^44^; **c** increasing the number of trials included in the average progressively stabilises gustatory evoked waveforms^105^; **d** conventional time-domain gERP averages show only subtle between-group differences, indicating that features beyond the canonical average should be extracted^39^; **e** stimulus-locked sweet-specific component: suppression of sweet taste reduces sucrose-evoked potentials relative to responses before suppression^105^.

In the time domain, gERPs provide a direct window onto when processing occurs, but their separation of taste identity and pleasantness is generally weak, whereas their support for intensity is moderate (Fig. 2). Relatively stable early components usually fall within the P1 time window at approximately 100–150 ms, and source-localisation and imaging results converge in pointing to rapid recruitment of the anterior insula and operculum within the gustatory network^32,33^. When stimulus intensity increases, ERPs often show increased amplitude or shortened latency. When taste is suppressed, for example by local anaesthesia, ERPs may also be correspondingly reduced or abolished^34–36^. Together, these findings support a moderate level of evidence for intensity modulation. However, variability in effective onset across trials, together with orofacial movement and somatosensory interference, usually yields low single-trial signal-to-noise, so early components are easily washed out after averaging. As a result, differences related to taste identity or pleasantness are difficult to observe stably in mean waveforms^37–39^. ERPs are therefore better suited to addressing when processing occurs than to serving as standalone evidence for the separation of gustatory attributes.

**Fig. 3:**
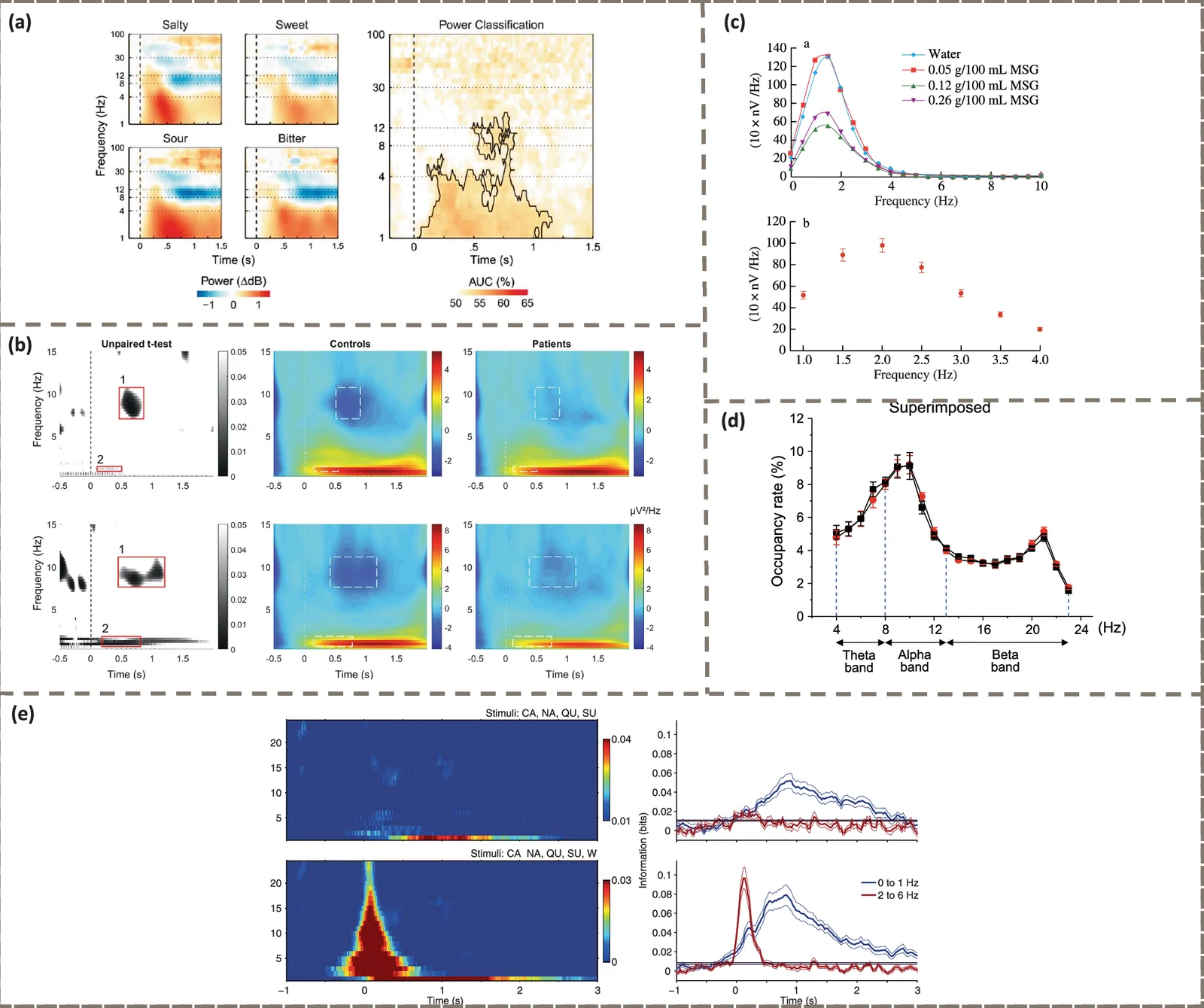
Evidence for frequency-domain feature-based discrimination. **a** Time–frequency analysis of EEG responses to different taste stimuli shows the highest energy in the delta-band^40^; **b** time–frequency analysis under the same gustatory stimulation in healthy participants and individuals with taste disorders reveals differences in the degree of delta-band activation^39^; **c** low-frequency differences induced by monosodium glutamate stimuli at different concentrations^41^; **d** low-frequency differences observed in a combined taste–olfaction paradigm under matched and mismatched gustatory–olfactory conditions^106^; **e** experimental evidence from mice supporting low-frequency encoding of gustatory information^107^.

In the frequency domain, the most substantial gain in observability comes from low-frequency activity, particularly the single-trial representation of taste identity in the delta-band (Fig. 3). Delta-band power and phase patterns can distinguish multiple taste categories within approximately 130 ms and closely match subjective recognition time. Classification is driven mainly by taste identity, is less strongly associated with intensity or subjective valence, and remains stable or even improves when the delta-band is isolated^39,40^. These results make delta activity one of the most consistent currently available signals for the detectability of taste identity. However, the interpretation of this evidence must remain more cautious than the statement of detectability itself. Low-frequency indices are especially vulnerable to slow drifts and movement-related confounds, such as swallowing, perioral electromyographic activity and respiration, all of which are common in gustatory paradigms. In addition, the existing literature does not describe swallowing monitoring, EMG recording and mitigation, matched tasteless or somatosensory or thermal control conditions or strict subject-wise validation in a standardised way. We therefore recommend that evidence for delta-band taste identity be described as strong for detectability but only moderate for interpretability. To make this point more transparent, we provide a structured supplementary summary table (Table S1) that summarises the extent to which representative delta-band studies addressed these confound-control criteria.

Low-frequency activity may also reflect differences in intensity or physiological state, such as differences across concentrations or between patient and control groups^41^. By contrast, evidence for theta and higher-frequency bands remains sparse in human gustatory EEG, and whether their modulation by attention or emotion can be strictly interpreted as reflecting pleasantness still lacks robust validation^42,43^. Thus, the key conclusion is not that one band is simply superior, but that gustatory information may be more robustly carried by low-frequency dynamics and phase organisation, while the extent to which these signals represent relatively pure gustatory information still depends heavily on the adequacy of confound control.

**Fig. 4:**
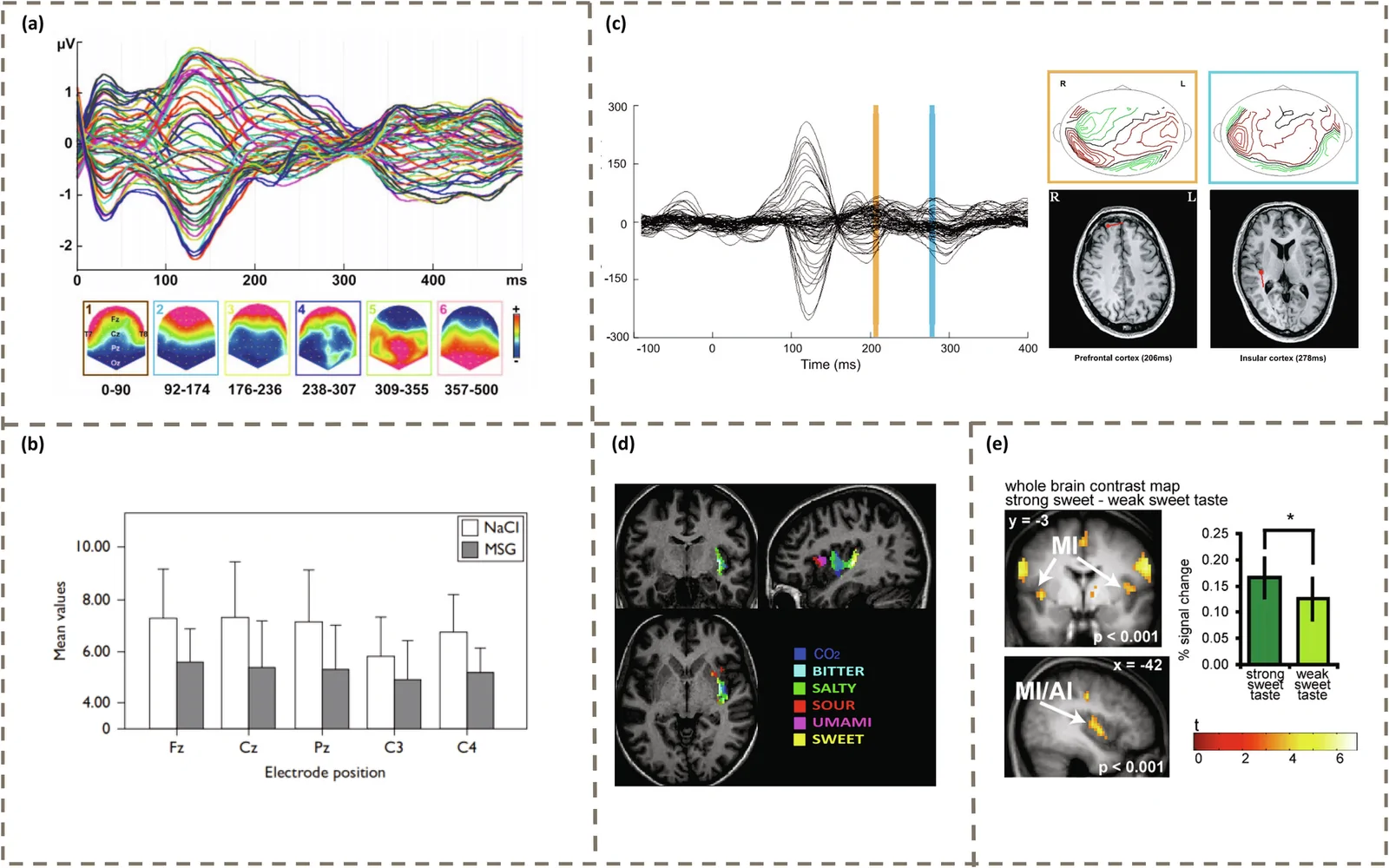
Evidence for spatial feature-based discrimination. **a** Polarity reversal in EEG waveforms^44^; **b** differential activation across taste conditions recorded from positionally symmetrical electrodes (C3 and C4)^108^; **c** polarity reversal observed in fMRI^109^; **d** brain maps showing the dominant cortical regions affected by different taste qualities in the right insular cortex^110^; **e** brain maps showing the effects of different taste intensities^111^.

At the spatial level, current findings provide relatively consistent support for an insula–operculum-centred source, but evidence that different tastes form stable and generalisable separation in scalp topography or source space remains limited (Fig. 4). Scalp topographies often show anterior–posterior polarity reversal, providing structural cues for multivariate spatial patterns^30,44^. Source reconstruction approaches more consistently localise P1-related activity to the anterior insula–operculum junction and can further extend to orbitofrontal cortex and anterior cingulate regions^33,45^. Some studies suggest subtle source-level differences for specific taste contrasts, such as salty versus umami, but the effects are usually small and often require additional support from fMRI for clearer differentiation^46,47^. Under conventional scalp EEG, stable spatial separation of taste identity or intensity generally requires high-density recordings together with multivariate methods, such as spatial-pattern classification or current source density algorithms^48,49^. Otherwise, only subtle differences are typically observed. For pleasantness, reproducible spatial-domain evidence is still lacking. Accordingly, the main role of spatial evidence is to provide interpretive constraint on source consistency rather than to guarantee attribute separability on its own.

**Fig. 5:**
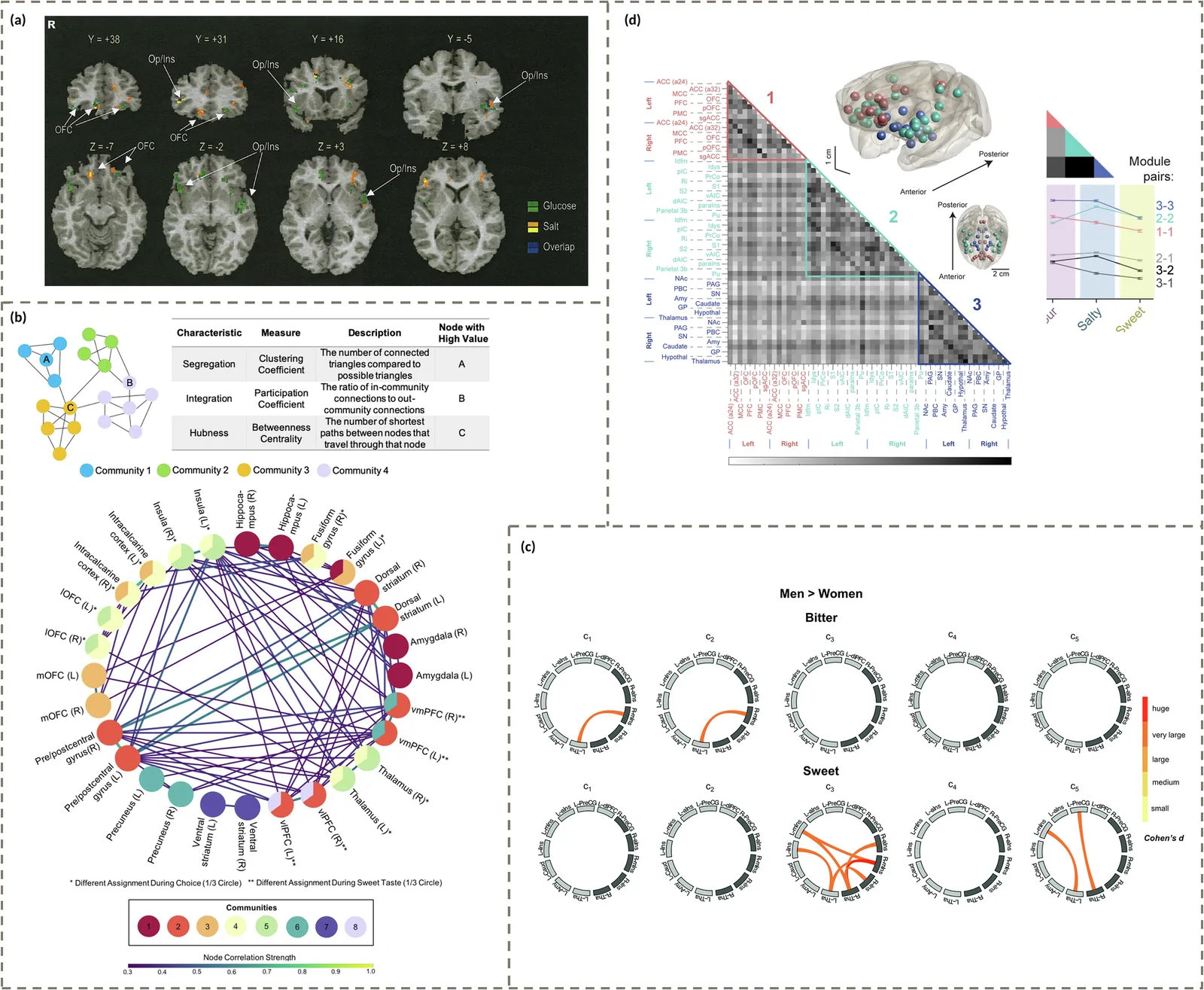
Evidence for connectivity-based discrimination. **a** Differences in spatial features alone are insufficient for fine-grained discrimination of gustatory properties, even when using higher-resolution fMRI^112^; **b** gustatory processing can be represented as a network of inter-regional connections, and this network-level organisation captures additional differences^51^; **c** different taste qualities can give rise to distinct connectivity patterns^50^; **d** the gustatory network is not an unstructured mass, but a system that can be organised into modules^52^.

In theory, network and connectivity features are more closely aligned with the distributed and coordinated nature of gustatory processing, but direct EEG evidence in this area remains weak, whereas stronger support currently comes mainly from fMRI and animal studies (Fig. 5). Integrative fMRI and graph-theoretic studies indicate that gustatory processing involves networks spanning the insula, thalamus, amygdala, striatum and frontal regions, and that connectivity strength and nodal importance vary with stimulus concentration or inter-individual differences^50,51^. On the EEG side, one suggestive clue is that cross-channel delta-phase patterns can discriminate taste identity, implying that gustatory stimulation may induce a coordinated multi-regional delta-phase pattern that can be understood as a functional-network representation^40,52^. Animal studies likewise suggest that different tastes alter network extent and modular organisation, and that the insula may serve as a hub-like node^51^. However, connectivity estimation is highly sensitive to data quality, temporal alignment and source leakage, and many studies focus only on activation maps, which are often insufficient to reveal interaction-level differences. Hypotheses such as enhanced insula–orbitofrontal synchrony for pleasant tastes still lack dedicated and reproducible human EEG evidence.

To improve reproducibility and interpretability in this area, future studies should move beyond coarse sensor-level summaries and adopt multidimensional analytical approaches that jointly consider space, time, frequency and phase^53^. In particular, source-space connectivity estimated using leakage-aware and bias-corrected metrics, such as the debiased weighted phase lag index (dwPLI) and source-leakage-aware amplitude envelope correlation (AEC), may reduce spurious coupling and improve inference. Likewise, high-resolution source-space analyses that use more vertices rather than only coarse atlas-based regions may reveal fine-grained functional architectures of taste processing that are currently overlooked^54^. These approaches may also help determine whether alpha- and beta-band network topographies and temporal dynamics contain complementary information beyond the detectability supported by the delta-band. Thus, the most appropriate current characterisation of network evidence is that it remains weak but is methodologically expandable, rather than that it already constitutes a primary source of evidence.

Taken together, examining gustatory EEG from a multi-feature perspective helps clarify how gustatory information is encoded across different dimensions and what limitations each dimension entails. For ease of comparison, Table 1 summarises the evidence strength across the four main feature domains.

Comparison across feature domains shows that no single EEG feature can fully characterise gustatory information, because each dimension comes with its own strengths and bottlenecks. Time-domain ERPs provide a temporal window onto gustatory processing, but the signals are weak and offer limited class separability. Frequency-domain analyses highlight the role of low-frequency oscillations and substantially improve effective signal-to-noise, but their strongest conclusions currently apply more to detectability than to fully confound-insensitive interpretability. Spatial features support the involvement of key structures such as the insula, but still offer limited separation among gustatory attributes. Network analysis remains at an early stage, yet suggests that some gustatory information may reside in patterns of interaction among brain regions and is therefore worth further investigation. Future work that integrates these dimensions in a complementary way may allow more reliable and more fine-grained extraction of gustatory information from EEG. In practice, if the aim is to improve observability of taste-related features, a reasonable priority is to use low-frequency time–frequency features, especially delta-band features, as the backbone, complemented by multivariate spatial patterns. If the focus shifts to pleasantness or contextual effects, greater emphasis should be placed on task design and network-connectivity indices in order to capture distributed interaction differences. This naturally leads to the next section, which treats decoding models as information probes and analyses how different signal structures support separability.

## Decoding models as information probes: interpretability and stringent validation

In gustatory EEG research, decoding models are often used to answer whether conditions can be separated. For non-invasive neuroimaging, however, the more critical question is why the model can separate them. Does it rely on gustatory representations themselves or on confounding cues such as movement, somatosensory input and expectation? And does the same conclusion remain valid across participants and paradigms? We therefore argue that decoding models should be positioned as information probes: the success or failure of different model structures reflects which separable cues are actually present in the signal. From this perspective, accuracy is not the endpoint, but only the starting point of an inference chain. It is important to emphasise that strong separability does not automatically imply strong mechanistic evidence. In gustatory EEG in particular, a model may show high detection performance while still offering weak interpretability because of insufficient confound control.

Traditional machine-learning models, such as linear discriminant analysis (LDA) and support vector machines (SVM), search for decision boundaries in a predefined feature space^55–57^. If stable separation can already be achieved using linear or near-linear models, this generally suggests that gustatory information has a relatively low-dimensional and near-linear separable structure in the chosen projection. If linear models consistently fail, whereas kernel methods or stronger non-linear models yield clear improvements, this more likely implies that the relevant information is embedded in higher-order interactions or non-linear manifolds and requires richer representation learning in order to become visible. Existing work does indeed support such a hierarchy of separability. Shallow approaches based on engineered features can achieve effective separation in some contrasts, such as DWT-based features combined with shallow networks or linear classifiers to separate sour and salty^58^, or LDA to distinguish sweet from tasteless^59^. However, the interpretability of such findings has a key prerequisite: swallowing, facial EMG and action strategies must be tightly controlled, otherwise apparent separability may simply reflect differences in task execution. For subtler distinctions, such as sucrose versus artificial sweeteners, simple frequency-domain features combined with SVM are often insufficient, whereas end-to-end convolutional neural networks (CNNs) can markedly improve discrimination^60,61^. This suggests that discriminative cues may reside in temporal dynamics or more complex patterns that cannot be fully captured by a small set of handcrafted linear features.

The value of deep models—including CNNs, recurrent neural networks (RNNs), long short-term memory networks (LSTMs), Transformers and graph convolutional networks (GCNs)—lies not only in improved performance, but also in the fact that their architectures embody different assumptions about coding^62–69^. CNNs tend to capture local spatiotemporal patterns within short time windows^70,71^. RNNs, LSTMs and Transformers are better suited to modelling long-range temporal dependencies and sequence evolution^72–74^. GCNs explicitly encode inter-channel relations, such as anatomical proximity or functional connectivity, as graph structure, thereby allowing direct tests of whether distributed coordination is necessary for successful discrimination^75^. Although graph models remain relatively rare in gustatory EEG, they have already shown clear advantages in modelling cross-channel relations in other cognitive tasks^76,77^, and therefore offer transferable methodological tools for gustatory research. At the same time, the more powerful the model, the more necessary it becomes to demonstrate that it is exploiting gustatory representations rather than procedural or artefactual cues. For this reason, comparisons among deep models should be accompanied by ablation analyses, such as removing high-artefact channels, shuffling trial order, or controlling intensity and movement cues, and by strict validation whenever the scientific claim concerns generalisable gustatory representations. Otherwise, apparent performance gains may simply reflect more efficient exploitation of confounds.

Accordingly, transforming model performance into valid evidence requires both a minimal interpretability chain and a stringent validation process. For linear or tree-based models, weights or feature-importance estimates can be used to identify which channels and frequency bands contribute most strongly to the decision. For example, studies that use LSBoost to predict coffee attributes can provide frequency-by-channel importance patterns, thereby turning what the model uses into a physiologically discussable clue^78^. For CNN-based models, class activation mapping, input perturbation or occlusion analyses can be used to localise critical time windows and channel sets and to test whether these cues cluster within plausible sensory-to-evaluative stages^79^. Further, low-dimensional visualisation of intermediate representations and cluster-stability analysis can be used to assess whether the organisation of different gustatory conditions in representation space is consistent with the structure of psychological dimensions^80–83^. When source-level interpretation is introduced, its credibility is much greater when supported by high-density EEG or MEG, individualised head models, anatomically constrained inverse solutions, and sensitivity analyses across source priors than when based on sensor-level decoding alone.

Validation standards are equally important. Information leakage—for example, when adjacent time windows from the same trial enter both training and test sets, or when participant splits are insufficiently strict—together with class imbalance and over-tuning can lead to inflated performance with poor reproducibility^83^. For gustatory EEG, strong decoding performance should ideally be reported together with the following information: whether swallowing was monitored or controlled; whether EOG or EMG were recorded and mitigated; whether tasteless or somatosensory or thermal control conditions were included; and whether strict subject-wise validation was adopted when claims of cross-subject generalisation are made. Some confusion patterns, such as sweet being harder to distinguish, whereas sour or bitter are easier, can indeed provide clues for external consistency checks. Even so, such patterns still require cautious interpretation in the light of confound control and interpretability evidence^84,85^.

For decoding models to function genuinely as probes of gustatory observability, a complete and rigorous inference chain is required. Within the revised framework, this chain can be summarised as follows: first, propose a structural hypothesis; then, assess changes in separability; next, conduct interpretable localisation analysis and representation-structure analysis; and finally, test the result using cross-subject generalisation together with ablation-based confound checks. In other words, decoding should not be viewed as a final accuracy report, but as a structured test of whether the proposed representation remains detectable, reproducible and interpretable under increasingly stringent constraints. Only when these components form a logical closed loop can decoding results be regarded as robust evidence that gustatory information is observable in scalp EEG, rather than as a performance number that is easy to overinterpret.

## Why observability remains low: from alignment to analysis bias

Low observability in gustatory EEG does not mean that gustatory information is absent. More commonly, information is systematically weakened along the chain of acquisition, alignment and modelling, resulting in poor detectability, weak reproducibility and limited interpretability. These limitations usually do not arise from a single source, but from the combined effects of several factors: uncertainty in stimulus timing and alignment, low signal-to-noise ratio together with deep-source projection limits, inter-individual variability and state fluctuations, and analytical heterogeneity together with statistical and validation biases. Understanding how these factors undermine the three core requirements of observability is the basis for building an effective improvement roadmap.

First, gustation suffers from a structural timing problem: valve triggering is not equivalent to subjective onset. The transport of liquid into the mouth, dissolution and diffusion, saliva mixing, and tongue-surface distribution all introduce non-negligible onset delay and trial-to-trial jitter^86^. This jitter weakens the consistency of early ERP peaks and reduces trial synchrony in phase and time–frequency measures, thereby compromising both detectability and interpretability. At the same time, gustatory delivery is inherently multimodal. Tactile, thermal and trigeminal inputs often accompany taste, while swallowing, tongue movement and respiration introduce systematic motor signals^87^. If the paradigm also includes immediate ratings or expectation manipulations, additional uncertainty in cognitive timing is introduced, making it even harder to determine whether the observed differences reflect gustatory representations or instead action and task load. This issue is especially important for low-frequency signals: delta-band effects may appear relatively stable at the level of detectability, but if temporal uncertainty and oral-motor confounds are not explicitly controlled, their interpretability may still be highly limited. Thus, even when genuine gustatory representations are present, the resulting effects may still appear unstable or difficult to interpret.

Second, low signal-to-noise ratio and deep-source projection limits form another hard constraint. Gustatory cortical sources are often located in deep Sylvian structures such as the insula and operculum, where depth attenuation, direction cancellation caused by cortical folding, and volume conduction all reduce net scalp potential amplitude. Meanwhile, gustatory paradigms are naturally prone to contamination from facial EMG, swallowing artefacts and ocular activity, thereby lowering single-trial signal-to-noise ratio^87,88^. Although increasing trial number can improve averaging, it also introduces adaptation, fatigue, attentional drift and threshold variation, producing a familiar trade-off: signal-to-noise may improve, but state-related noise also increases, so detectability and reproducibility do not improve linearly^34^. This is one reason why some features may appear relatively robust in averaged waveforms or classifier outputs, while still falling short of strong interpretability standards.

Third, inter-individual variability and state fluctuation transform the observability problem from a signal problem into an inference problem^89–92^. Taste thresholds, preferences, experience, salivary secretion and action strategies can all alter the amplitude and timing of brain responses. Hunger or satiety, attentional level, expectation and emotion can also introduce pronounced drift within the same participant across sessions^93^. In addition, EEG is affected by variation in electrode placement, reference choice and head models, reducing the cross-subject consistency of spatial patterns. If such variability is treated simply as noise—for example, by performing hard averaging across participants or by training cross-subject models without any domain adaptation—the typical outcome is a situation in which data are decodable within participants but fragile across participants. This directly weakens reproducibility and prevents cumulative evidence from emerging. In this sense, reproducibility depends not only on signal quality but also on experimental design and validation logic.

Fourth, analytical heterogeneity together with statistical and validation bias can make methodological differences appear as physiological differences. Even when raw data are similar, different choices in filtering, referencing, baseline correction and artefact handling can substantially alter ERP and time–frequency results^94–96^. Different time-window selection and feature-engineering choices can also change observability estimates and reduce comparability across studies. Statistical practice is equally critical. Selective reporting under multiple comparisons increases false positives, while cross-validation leakage—for example, when adjacent windows from the same trial enter both the training and test sets, or when participant splits are not sufficiently strict—together with class imbalance and excessive hyperparameter tuning can produce inflated performance that is not reproducible^83^. In gustatory EEG, this means that strong classifier performance does not automatically answer the question of observability. If swallowing control, EMG and EOG mitigation, matched control conditions and participant-level validation are not clearly reported, high performance may still reflect detectability that is highly sensitive to confounds rather than an interpretable gustatory representation. Under such circumstances, what is truly scarce is not a more complex model, but a validation chain that is interpretable, auditable and physiologically grounded.

Overall, the first two groups of factors—uncertain timing and low signal-to-noise ratio—primarily erode stable detectability, whereas the latter two groups—inter-individual variability and analytical bias—primarily erode reproducibility; all four together reduce interpretability. In other words, gustatory EEG often fails not because gustatory information is absent, but because the evidence is compressed, distorted or confounded before it can become cumulative. Improving observability, therefore, requires not only better signal extraction but also stricter paradigm control, more systematic confound accounting, and more credible validation logic. The next section integrates the preceding evidence and translates these constraints into a best-practice roadmap for cumulative evidence.

## Conclusions and outlook

Using gustation as a prototypical low-observability modality, this review advances a central claim for non-invasive neuroimaging: in EEG, it is not enough simply to determine whether an effect exists; we must also determine what level of observability that effect reaches, where the bottlenecks lie, and how evidence can accumulate. At the level of neural pathways and signal generation, cortical nodes such as the insula–operculum complex are more likely to produce measurable scalp potentials, whereas deep relays are more likely to influence EEG indirectly by modulating cortical input. At the evidential level, ERPs provide moderate support for intensity-related change, but overall offer limited and inconsistent separation for taste identity and pleasantness. In the frequency domain, delta-band activity currently provides one of the most consistent signals for the detectability of taste identity, although its interpretability still depends strongly on the adequacy of confound control. Spatial and network indicators are better regarded as interpretive constraints and as components of a next-stage evidence chain, but their robustness and generalisability still depend on high-density recordings, refined head models, leakage-aware estimation and consistent validation standards. At the inferential level, decoding models should be regarded as information probes: only when structural hypotheses, interpretable localisation and stringent validation form a coherent loop do performance results acquire genuine mechanistic meaning.

On the basis of these conclusions and the limitations identified above, we propose a minimal but actionable best-practice checklist. Its core principle can be summarised as follows: standardised workflows as the foundation, complementary feature integration as the gain, and interpretability together with stringent validation as the source of credibility.

The first priority is to reduce temporal jitter and improve event definition. This requires brief stimuli, fixed and controllable inter-stimulus intervals, standardised rinsing procedures, explicit recording of delivery timing, and efforts to minimise the gap between valve triggering and effective stimulus onset. Whenever feasible, swallowing, tongue movement and respiration should be monitored or controlled. Tasteless conditions as well as matched somatosensory and thermal control conditions should also be included in order to estimate non-gustatory contributions and to separate signal detectability from interpretability that remains vulnerable to confounds.

The second priority is to improve signal quality and strengthen confound control. Recording quality and stable electrode impedance should be prioritised. High-density EEG should be adopted whenever possible in order to support source localisation and connectivity analysis. EOG should be recorded, and EMG should be recorded where feasible, together with clearly specified and reviewable artefact-processing procedures. In interpreting the results, neural signals must be distinguished from oral-motor, ocular and other physiological confounds, rather than being treated as a single undifferentiated low-frequency component.

The third priority is to choose features and models on the basis of complementarity rather than complexity alone. Low-frequency time–frequency features, especially those in the delta-band, together with spatial patterns, can form the backbone of evidence for taste identity. ERPs and low-frequency indices should instead be treated as complementary evidence for taste intensity. For pleasantness, interpretable contrasts should mainly be sought through task design rather than through model complexity alone. When network-level analyses are conducted, studies should increasingly move towards source-space estimation, leakage-aware or leakage-corrected measures and multidimensional analyses that jointly consider space, time, frequency and phase.

The fourth priority is to improve the reproducibility of connectivity and network claims. Future work should move beyond coarse sensor-level summaries whenever possible and give priority to source-space connectivity analysis together with leakage-aware or bias-corrected metrics such as dwPLI and source-leakage-aware AEC. Graph-construction choices, threshold sensitivity and frequency specificity should also be reported explicitly. Compared with coarse atlas-based parcellations, high-resolution source-space analyses using a larger number of vertices may help reveal fine-grained functional architectures of gustatory processing that are currently overlooked^53,54^. These approaches may also clarify whether the network topologies and temporal dynamics of alpha and beta bands provide information beyond the detectability offered by the delta-band.

The final priority is to enforce stringent validation and transparent reporting. Strict subject-level validation, prevention of information leakage, nested cross-validation when needed, explicit reporting of class-imbalance handling, hyperparameter-search ranges, random seeds and key parameters, and the sharing of code and critical processing scripts whenever possible should all be regarded as minimum standards rather than optional improvements. For studies making strong claims about taste-identity decoding, it is especially important to report explicitly whether swallowing monitoring and control were implemented, whether EMG and EOG were recorded, whether matched control conditions were included, and whether confound-oriented ablation analyses were performed.

This checklist is not an idealised wish list. On the contrary, it is a practical requirement if gustatory EEG is to move from scattered findings towards a comparable and cumulative evidence base.

At the application level, two broad pathways can be distinguished. The first is already feasible under current signal-quality conditions and includes objective assessment and subtyping of taste dysfunction, in which time–frequency indices may help distinguish patient and control groups^97^. In consumer and food contexts, EEG-based prediction of perceptual attributes such as bitterness, sweetness, sourness and astringency has already shown practical promise^98^ and may further support cross-modal consistency testing, sensory evaluation and formulation optimisation^99–102^. The second pathway depends on further improvement in observability, especially robust single-trial decoding with effective confound separation. This includes closed-loop neurofeedback for eating behaviour, such as weight management or preference modulation^103^, as well as integration with immersive systems for taste reproduction and remote tasting experiences, such as e-Taste^104^. In both pathways, credibility rests on the same foundation: if it cannot be shown that the model depends on gustatory neural representations rather than swallowing, EMG, expectation or other non-gustatory cues, then high performance is not merely unreliable, but may also introduce systematic bias into clinical, consumer-science or engineering decisions.

More broadly, the challenges that make gustation a low-observability modality are not unique to taste. Deep or folded cortical generators, strong movement-related confounds, uncertain temporal alignment and substantial inter-individual variability are also present in interoceptive and natural-behaviour research. The observability framework and best-practice checklist proposed here, therefore, have wider methodological significance. They provide a path from separability to interpretable and cumulative evidence, allowing future studies not only to ask whether decoding is possible, but also to ask what such decoding means in neurobiological terms.

## Supplementary information

Supplementary Table S1

## Data Availability

No datasets were generated or analysed during the current study.
