# Supplementary material for: Observability of gustatory information in scalp EEG
Source: NPJ Sci Food. 2026 Jul 7;10:334. doi: 10.1038/s41538-026-00957-7 (PMC13720129; doi:10.1038/s41538-026-00957-7)
Supplement: Supplementary file 1 [file 41538_2026_957_MOESM1_ESM.pdf]

Table S1: **Confound-control summary for representative low-frequency gustatory EEG studies cited in Section 3**

Study and manuscript ref.	Primary relevance in this Review	Human scalp EEG	Swallowing monitored or controlled	EOG recorded and mitigated	EMG recorded and mitigated	Matched tasteless control	Matched somatosensory or thermal control	Strict subject-wise validation	Main interpretive note	Main caveat
**Wallroth et al. 2018**	Direct single-trial taste identity decoding in the delta band	Yes	NR	NR	NR	NR	NR	NR	Strongest directly relevant human EEG study for delta-based identity detectability in the cited set	Detectability is strong, but interpretability remains confound-sensitive unless physiological controls and validation details are explicitly documented.
**Mastinu et al. 2024**	Low-frequency differences between healthy participants and patients with taste disorders	Yes	NR	Yes	NR	No	No	N/A	Supports low-frequency sensitivity to clinical or state differences rather than direct identity decoding	Better artefact transparency than most studies, but swallowing and EMG control are not explicitly reported in the accessible methods.
**Wu et al. 2022**	Low-frequency response to MSG concentration	Yes	Partial	NR	NR	Partial	No	N/A	Useful for low-frequency sensitivity to concentration or intensity	Supports low-frequency modulation, but not identity-specific inference; physiological confound control is limited in the accessible methods.
**Maeda and Yoshimura 2019**	Low-frequency effect in a matched versus unmatched odor–taste paradigm	Yes	NR	Partial	NR	No	No	N/A	Informative for cross-modal low-frequency modulation	Relevant to context-dependent modulation rather than pure taste-identity decoding; explicit swallowing and EMG control are not reported.

Legend: Yes=explicitly reported; Partial=some relevant control reported, but not fully matched to the criterion; NR=not clearly reported in the accessible text; N/A=not applicable to the main analytic claim.
